# Infant sleep EEG features at 4 months as biomarkers of neurodevelopment at 18 months

**DOI:** 10.1038/s41390-025-03893-6

**Published:** 2025-02-20

**Authors:** Soraia Ventura, Sean R. Mathieson, John M. O’Toole, Vicki Livingstone, Deirdre M. Murray, Geraldine B. Boylan

**Affiliations:** 1 INFANT Research Centre, University College Cork, Cork, Ireland; 2 Department of Paediatrics & Child Health, University College Cork, Cork, Ireland

## Abstract

**Background:**

Sleep parameters evolve in parallel with neurodevelopment. Sleep participates in synaptic homeostasis and memory consolidation and infant sleep parameters correlate with later aspects of early childhood cognition.

**Methods:**

Typically developing, term-born infants had a diurnal sleep-EEG at 4 months and Griffiths III developmental assessment at 18 months. EEG analysis included sleep macrostructure (i.e. durations of total sleep and sleep stages, and latencies to sleep and REM), sleep spindle features, and quantitative EEG features (qEEG): interhemispheric connectivity and spectral power. We assessed the correlations between these EEG features and Griffiths III quotients.

**Results:**

Sleep recordings from 92 infants were analyzed. Sleep latency was positively associated with the Griffiths III Foundations of Learning subscale and N3 sleep duration was positively correlated with the Personal-Social-Emotional subscale. Sleep spindle synchrony was negatively associated with Eye and Hand Coordination, Personal-Social-Emotional, Gross Motor, and General Development quotients. Sleep spindle duration was negatively associated with the Personal-Social-Emotional and Gross Motor subscales. In some sleep states, delta 1 and 2 EEG spectral power and interhemispheric coherence measures were correlated with subscale quotients.

**Conclusion:**

Certain sleep features in the EEG of 4-month-old infants are associated with neurodevelopment at 18 months and may be useful early biomarkers of neurodevelopment.

**Impact:**

- This study shows that the EEG during infant sleep may provide insights into later neurodevelopmental outcomes.
- We have examined novel EEG sleep spindle features and shown that spindle duration and synchrony may help predict neurodevelopmental outcomes.
- Sleep macrostructure elements such as latency to sleep, N3 duration, and qEEG features such as interhemispheric coherence and spectral power measures at 4 months may be useful for the assessment of future neurodevelopmental outcomes.
- Due to exceptional neuroplasticity in infancy, EEG biomarkers of neurodevelopment may support early and targeted intervention to optimize outcomes.

## Introduction

In childhood, several critical periods of development, characterized by an enhanced environment-dependent sensitivity for the establishment of synaptic connectivity, promote long-lasting skill or trait acquisition.^1^ Notably, some essential critical periods associated with vision, auditory and language acquisition, and the development of intra- and interpersonal skills occur during the early years.^1,2^ Early identification of atypical trends in neurodevelopment may enable targeted intervention to help improve longer-term outcomes.^3^ To facilitate standardized assessment, and to facilitate the detection of atypical developmental trajectories, the psychologist Dr Ruth Griffiths developed the *Baby Scales*,^4^ first published in 1954, which resulted from a meticulous review of the behavior of infants and young children throughout the first years of life. Successive observations resulted in the characterization of stepwise milestones across locomotor, personal-social, hearing and speech, eye and hand coordination, and performance areas. These studies provided the framework for the most recent Griffiths Developmental Scales: Griffiths Scales of Child Development, third edition (Griffiths III).^5^

**Fig. 1:**
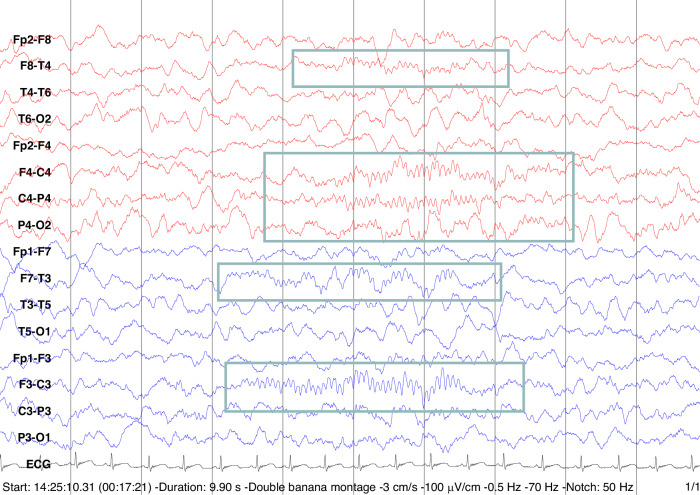
Sleep spindles of a 4-month-old. Sleep spindles (in grey boxes) are transient waveforms of sleep occurring as runs of 11–16Hz^54^ activity during N2 and N3. Vertical lines represent 1-second intervals.

The first years of life, particularly infancy, represent exceptionally active periods of neurodevelopment. The brain doubles in volume, primarily due to the pronounced expansion of grey matter, reflecting both dendritic growth and the establishment of novel synapses^6,7^ with concurrent pruning of superfluous connections.^8^ Intense myelination^9^ and functional changes take place.^10,11^ Some of these functional changes can be assessed through sleep EEG. Sleep is ideal for studying existing EEG patterns with reduced influence from external inputs and is also integral to normal brain functioning^12,13^ and maturation.^14^ A meta-analysis on pediatric sleep duration (almost exclusively on studies during night-time sleep) shows weak associations with several cognitive and behavioral domains.^15^ Meta-analyses of the relationship between sleep and cognitive features (memory and sustained attention) show different results in children and adults, possibly reflecting methodological and neurobiological differences.^15^ While sleep duration partially reflects some aspects of cognitive ability, other sleep features are important markers of outcome. Specific sleep patterns reflect and/or actively participate in brain maturation and cognitive ability,^16–21^ supporting associations between learnt concepts^22^ and, neurodevelopment.^23–30^ For example, in early life, functional connectivity, anatomical networks, and sleep evolve in tandem in an interlinked process.^31^ Neonatal changes in functional connectivity between sleep states in the posterior region may modulate visual network development, associated with visual performance at the age of 2 years.^32^ Slow waves during NREM (non-rapid eye movement) sleep have been associated with homeostatic processes and synaptic down-selection,^12,13^ with particular relevance during maturation^24^; and properties of this slow rhythm such as anterior-posterior ratio and propagation are associated with myelination and anatomical brain connectivity,^28,33^ and future skill learning.^26^ Beaugrand et al. however, described statistically non-significant results between anterior-posterior ratio at 6 months and development assessment scores; one possible explanation may be related to the relatively homogeneous cohort.^34^ The topographic distribution of sleep spindles (Fig. 1) and other spindle characteristics are also related to previous learning activities,^20,35–39^, and appear to influence mental abilities^17–19,40^ and sleep maintenance.^41,42^ Spindles in infancy are also believed to be involved in somatosensory mapping.^25^ Both sleep spindles and slow wave co-action appear to contribute to memory consolidation.^43,44^ Despite all this evidence, comparisons between studies analyzing sleep patterns and cognitive/neurodevelopmental trajectories are complicated by the use of different methodologies, different age groups, and generally low sample sizes.^29,30,45,46^

Sleep spindles,^47,48^ spectral power^49,50^, and coherence^49,51,52^ change during brain maturation. qEEG features change dynamically with the sleep stages, therefore, qEEG features are analyzed for each sleep stage separately.^31,53^ The sleep spindle features included in our analysis are those identified by Louis et al.^47^ as showing a trend or statistically significant changes during the first six months after birth.^47^ Acknowledging that neurodevelopment is a continuous process shaped by environment and biology, this exploratory study aims to define the key sleep EEG features at 4 months associated with developmental outcomes at 18 months for future validation. Considering the studies discussed above, we hypothesize that sleep spindle features and spectral power, particularly in the delta range, and coherence are associated with Griffiths III General Development (GD) and subscales at 18 months.

## Methods

### Cohort

Infants born at or after 37 weeks of gestation were recruited at Cork University Maternity Hospital between 2017 and 2018 as part of the BabySMART (Study of Massage Therapy, Sleep, and Neurodevelopment, registered identifier NCT03381027) study, a randomized controlled trial on sleep and massage intervention, approved by the Research Ethics Committee of the Cork Teaching Hospitals. The infants were singletons, not requiring admission to the neonatal unit, and without suspected congenital or metabolic conditions. A total of 408 infants were enrolled in the BabySMART study, of which half (*n* = 204) were randomized to the non-intervention group. Only data from infants in the non-intervention arm was analyzed in this study and, to be included, infants had to have a sleep EEG recorded at approximately 4 months and the Griffiths III at 18-months. A total of 92 infants met these criteria and were included in the final analysis. The participant’s parents or guardians provided written informed consent and the study was approved by the Clinical Research Ethics Committee (CREC) of the Cork Teaching Hospitals.

### EEG recording and analysis at 4 months

EEG was recorded using the Lifelines iEEG (Lifelines Neuro, UK) system when infants were circa 4-months. The EEG electrodes included Fp1, Fp2, Fz, F3, F4, F7, F8, Cz, C3, C4, T3, T4, Pz, P3, P4, T5, T6, O1, O2, A1, A2, reference and ground, placed according to the 10-20 International System; impedances were maintained below 10 kΩ. The polysomnographic channels ECG, EOG, chin EMG, and a movement sensor to record respiration were also applied. Appointments took place at the sleep lab of the INFANT Center, University College Cork, Ireland during the daytime. Sleep was recorded during conditions of low noise and dimmed lights; the recording started once all electrodes were applied and ended when the infant was fully awake. A clinical physiologist remained in the room to ensure optimum technical quality of the EEG throughout the recording.

**Fig. 2:**
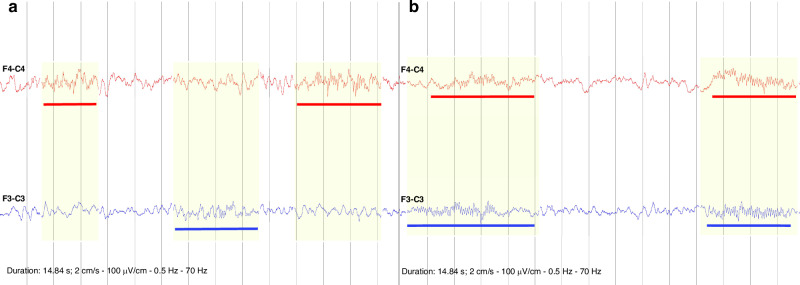
Sleep spindle synchrony. **a** example of low synchrony; right (in red) and left (in blue) frontocentral spindles do not coincide in time. **b** example of high synchrony; bilateral spindles overlap in time for the majority of their duration. Vertical lines represent 1-second intervals.

Sleep staging was performed using the Nicolet (Natus, Middleton, Wisconsin) sleep staging software according to the American Academy of Sleep Medicine (AASM) 2.4 guidelines.^54^ Sleep macrostructure refers to the parameters derived from sleep staging; we analyzed total sleep time, duration of sleep stages N1, N2, N3, and REM (rapid eye movement), sleep duration during the first cycle, and latencies to sleep (period from lights off to sleep onset) and REM (period from sleep onset to REM onset). Sleep spindles were visually identified and annotated from the left and right fronto-central EEG channels. Spindle number and duration were then directly extracted from the annotation list of the EEG review software (Stratus EEG, Reykjavík, Iceland). Sleep spindle density was defined as the number of spindles per minute in NREM sleep during the first sleep cycle. Mean sleep spindle frequency, sleep spindle synchrony (calculated as the duration of overlap in both fronto-central channels, divided by the total spindle time for both channels, expressed as a percentage, Fig. 2), brain symmetry index (BSI)^55^ and spectral power were calculated for sleep spindles. Quantitative EEG (qEEG) features were calculated on the continuous EEG, namely spectral power, and interhemispheric coherence. These features were calculated across all bipolar channels over the first sleep cycle for each sleep stage. Spectral power and global interhemispheric coherence were calculated for 7 frequency bands: delta 1, 0.5–2 Hz; delta 2, 2–4 Hz; theta, 4–8 Hz; alpha, 8–12 Hz; sigma, 12–15 Hz; beta, 15–30 Hz, gamma, 30–45 Hz. All qEEG features were calculated in MATLAB (The MathWorks Inc, Natick, Massachusetts, version 9.8.0 R2020a). Spectral power and global interhemispheric coherence were generated using the NEURAL toolbox^56^ for MATLAB. For further details on the sleep spindle and quantitative EEG (qEEG) analyses, please refer to Ventura et al.^57,58^

With the exception of total nap duration, sleep spindles, macrostructure, and qEEG parameters were based exclusively on the first sleep cycle to avoid potential variability due to the reduction of sleep pressure across successive cycles. Other macrostructure and qEEG features analysis included only infants with a complete first sleep cycle recorded, i.e., infants who fell asleep after the recording started and had both NREM and REM recorded during the first sleep cycle. Infants who fell asleep before the recording started were also excluded from sleep spindle number and density analyses.

### Developmental outcomes at 18 months and sociodemographic characteristics

Infants were assessed at the sleep lab using Griffiths III at 18 months, administered by a clinical neuropsychologist or pediatrician with training from the Association for Research in Infant and Child Development (ARICD). The Griffiths III is a tool that assesses pediatric development progress and flags possible learning disorders at an age range from birth up to 6 years. It has been recently re-standardized for UK and Irish pediatric populations.^5^ Griffiths III assesses 5 domains of development: A – Foundations of Learning, B – Language and Communication; C – Eye and Hand Coordination; D – Personal-Social-Emotional and E – Gross Motor. Subscale A evaluates aspects of learning such as executive function, working memory, and play. Subscale B evaluates expressive and receptive language; in preverbal children, this subscale evaluates children’s engagement with auditory stimuli and intention to communicate. Subscale C evaluates aspects that include fine motor and visuo-perceptual skills. Subscale D concerns the growing understanding of the concept of self, social interactions, and emotional responses. Subscale E assesses parameters like body coordination and strength. The GD score is derived from the average of all subscale scores. The developmental quotient of the infant of each subscale and GD scores were calculated for each infant. The developmental quotients are reported to have a mean (SD) of 100(15).^5^ Lower quotients on the subscales and overall scale indicate developmental delay. The upper and lower thresholds of the Griffiths quotients are >150 and <50, respectively. No exclusions were performed based on the Griffiths III quotients as we aim to investigate the relationship between sleep features and developmental outcomes for all infants.

Sociodemographic characteristics were described using information collected at study registration, as well as data from the 2-week and 4-month parent-completed questionnaires. Maternal-infant postnatal attachment at 4 months was measured using the 19-item Maternal Postnatal Attachment Scale.^59^ Scores can range from 19 to 95, with higher scores representing greater maternal-infant attachment. Maternal postnatal depression at 4 months was measured using the 10-item Edinburgh Postnatal Depression Scale (EPDS).^60^ Scores can range from 0 to 30 with higher scores representing a greater likelihood of depression.

### Statistical analysis

All statistical analyses were performed using IBM SPSS Statistics (version 26.0, IBM Corp., Armonk, New York). Spearman’s rank correlation coefficient was used to investigate and quantify relationships between the 4-months sleep features and Griffiths III developmental quotients at 18-months. Partial Spearman’s rank correlation coefficients were used to adjust for gestational age, ages at EEG and Griffiths III assessments, and sex. Socioeconomic factors, measured by maternal level of education and household income, were considered to have potential confounding effects on the relationships between sleep features and Griffiths III subscale quotients. To investigate this, associations between the sleep features, Griffiths III subscale quotients and socioeconomic factors were assessed. When both a sleep feature and a Griffiths III subscale quotient were associated with the same socioeconomic factor, a secondary analysis was performed calculating the Partial Spearman’s Rank Correlation between the sleep feature and Griffiths III subscale quotient, adjusting for the previous confounders and the socioeconomic factor. Absolute correlations less than 0.1 represent a very small association, coefficients between 0.10 to 0.29 represent a small association, coefficients between 0.30 to 0.49 represent a medium association and coefficients of 0.5 and above represent a large association.^61^ Multiple hypothesis testing of correlation coefficients was not adjusted for in the analysis as the study was exploratory in nature and because reducing the probability of a type I error would increase the probability of a type II error.^62,63^ Multivariable analysis was not performed due to the large number of independent variables investigated and the relatively small sample size.

## Results

### Demographics

**Fig. 3:**
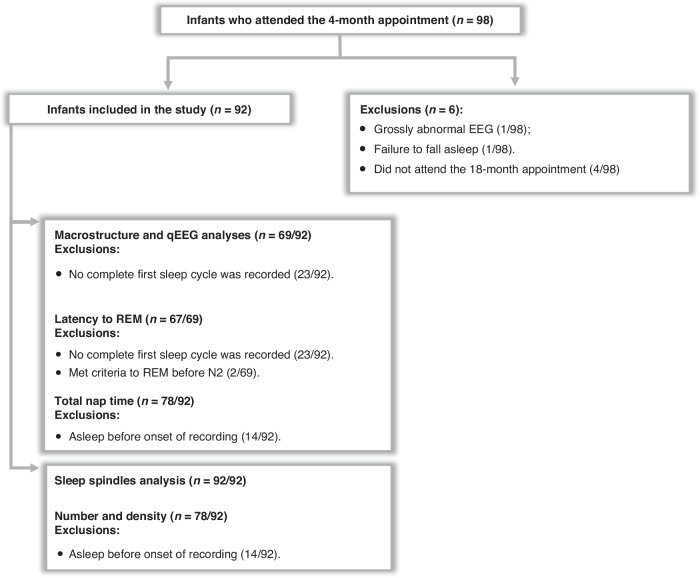
Study flow chart. Summary of recruitment numbers and exclusion factors.

Of the 204 infants randomized to the non-intervention group, 98 attended the 4-month EEG appointment, and 96 had a non-pathologic sleep EEG recorded (one infant did not fall asleep during the EEG, and another had a grossly abnormal EEG and was referred for further clinical assessment). Four infants were unable to attend the 18-month Griffiths appointment within the requested time period. Therefore, 92 infants were included in this study (Fig. 3). Table 1 presents the demographic characteristics of the infants, 40% of whom were female (*n* = 37). The median (interquartile range: IQR) postnatal age was 4.4 (4.3 to 4.7) months (range: 3.9 to 5.1 months) and 18.4 (18.2 to 18.7) months (range: 17.8–19.8 months), at the 4- and 18-months appointments respectively (*n* = 92). The 4-month EEG recordings had a median (IQR) total duration of 65.2 (49.5–87.9) minutes (range: 27.5 to 159.0 minutes) (*n* = 92). Table 2 presents the median (IQR) and observed range of Griffiths quotients at 18 months.

**Table 1:** Demographics, *n* = 92.

Demographics	*n*(%)^a^
**Registration and 2-week questionnaire**	
Sex – Female	37 (40.2)
Gestational age (weeks), mean (SD)	39.8 (1.2)
Weight at birth (Kg), mean (SD)	3.57 (0.46)
Maternal age (years), mean (SD)	35.1 (4.1)
Household net income/annum	
≤€30,000	7 (7.6)
€30,001-60,000	19 (20.7)
≥€60,001	39 (42.4)
Prefer not to say/ do not know	27 (29.3)
Highest level of education	
Secondary school	7 (7.6)
Third level certificate or diploma	17 (18.5)
Degree or higher diploma	36 (39.1)
Postgraduate qualification	32 (34.8)
**4-month appointment and questionnaire**	
Feeding at 4 months	
Breast Fed	26 (28.3)
Infant Formula	53 (57.6)
Both	13 (14.1)
Maternal attachment at 4 months^b^, median (IQR)	86.8 (82.7–90.6)
Maternal EPDS at 4 months^c^, median (IQR)	4 (1–8)

^a^Unless otherwise stated.

^b^Measured using the Maternal Postnatal Attachment Scale.^59^ Scores can range from 19 to 95, with higher scores representing greater maternal-infant attachment.

^c^Measured using the Edinburgh Postnatal Depression Scale.^60^ Scores can range from 0 to 30 with higher scores representing greater likelihood of depression.

**Table 2:** Neurodevelopmental outcome at 18 months - Griffiths developmental quotients (DQ), *n* = 92

Griffiths III	Median (IQR)	Observed range	
		Minimum	Maximum
Subscale A DQ, Foundations of Learning	114 (105–122)	68	130
Subscale B DQ, Language and Communication	109 (99–117)	67	145
Subscale C DQ, Eye and Hand Coordination	118 (109–122)	93	150
Subscale D DQ, Personal-Social-Emotional	122 (117–125)	88	132
Subscale E DQ, Gross Motor	135 (126–135)	50	150
GD DQ, General Development	122 (112–127)	84	141

Upper and lower thresholds of the Griffiths III quotients are >150 and <50, respectively. Low quotients indicate developmental delay.

### Sleep Macrostructure

Correlations between sleep macrostructure features at 4-months and Griffiths developmental quotients at 18-months are presented in Table 3. In the unadjusted analysis, a small positive association was observed between latency to sleep and the Foundations of Learning subscale. There was also a positive association between N3 duration and the Personal-Social-Emotional subscale. After adjustment, these associations remained significant and increased slightly in magnitude. A small negative association was observed between N2 duration and the Personal-Social-Emotional subscale in the unadjusted analysis, but this association was no longer statistically significant in the adjusted analysis.

**Table 3:** Association between macrostructure features and Griffiths developmental quotients at 18-months, *n* = 69^a^.

Griffiths scales/ macrostructure features (min)	Subscale A DQ, Foundations of Learning				Subscale B DQ, Language and Communication				Subscale C DQ, Eye and Hand Coordination				Subscale D DQ, Personal-Social-Emotional				Subscale E DQ, Gross Motor				GD DQ, General development			
	Spearman’s Rank Correlation		Partial Spearman’s Rank Correlation^b^		Spearman’s Rank Correlation		Partial Spearman’s Rank Correlation^b^		Spearman’s Rank Correlation		Partial Spearman’s Rank Correlation^b^		Spearman’s Rank Correlation		Partial Spearman’s Rank Correlation^b^		Spearman’s Rank Correlation		Partial Spearman’s Rank Correlation^b^		Spearman’s Rank Correlation		Partial Spearman’s Rank Correlation^b^	
	r	p	r	p	r	p	r	p	r	p	r	p	r	p	r	p	r	p	r	p	r	p	r	p
Total nap duration^c^	‒0.07	0.57	‒0.11	0.37	‒0.03	0.77	‒0.08	0.51	‒0.14	0.22	‒0.19	0.11	‒0.13	0.27	‒0.18	0.13	‒0.03	0.81	‒0.06	0.61	‒0.08	0.47	‒0.15	0.20
Duration N1	‒0.003	0.98	‒0.002	0.99	‒0.08	0.49	‒0.13	0.32	0.05	0.67	0.05	0.72	‒0.01	0.94	‒0.04	0.76	0.04	0.72	0.04	0.76	0.08	0.54	0.08	0.55
Duration N2	‒0.07	0.59	‒0.04	0.73	‒0.23	0.06	‒0.18	0.16	‒0.22	0.07	‒0.17	0.17	‒0.29*	0.02	‒0.23	0.07	‒0.06	0.60	‒0.02	0.88	‒0.14	0.26	‒0.10	0.45
Duration N3	0.07	0.59	0.09	0.49	0.01	0.94	0.02	0.89	0.09	0.48	0.12	0.36	0.24*	0.05	0.27*	0.03	0.09	0.46	0.10	0.44	0.04	0.72	0.08	0.55
Duration REM	‒0.08	0.53	‒0.11	0.39	0.03	0.79	‒0.01	0.94	‒0.08	0.54	‒0.14	0.27	‒0.11	0.37	‒0.17	0.17	0.03	0.79	0.01	0.91	‒0.01	0.91	‒0.08	0.55
Latency to sleep	0.27*	0.02	0.28*	0.03	‒0.04	0.78	‒0.05	0.68	0.06	0.66	0.04	0.73	0.10	0.40	0.10	0.45	0.04	0.75	0.04	0.75	0.06	0.64	0.04	0.74
Latency to REM^d^	‒0.06	0.65	‒0.03	0.81	‒0.16	0.19	‒0.18	0.15	0.10	0.41	0.12	0.33	0.10	0.44	0.10	0.42	0.06	0.65	0.07	0.59	0.04	0.77	0.05	0.69

^*^Coefficients whose *p*-value < 0.05.

^a^Unless otherwise stated.

^b^Partial Spearman’s Rank Correlations are adjusted for postnatal age at both EEG and Griffiths assessments, gestational age, and sex.

^c^*n* = 78, infants who fell asleep during EEG recording.

^d^*n* = 67, infants who had a full sleep cycle recorded and reached REM at the end of it.

### Sleep Spindles

Correlations between sleep spindle features at 4-months and Griffiths III developmental quotients at 18 months are presented in Table 4. Increased sleep spindle synchrony percentage was associated with lower quotients in the general development scale and three subscales; Eye and Hand Coordination, Personal-Social-Emotional and Gross Motor subscales. The strongest association was with the Gross Motor subscale. These associations increased slightly after adjustment for gestational age, ages at the assessments, and sex. Increased spindle duration was associated with decreased quotient values in both the Personal-Social-Emotional and Gross Motor subscales and these associations remained significant in the adjusted analysis.

**Table 4:** Association between sleep spindle features and Griffiths developmental quotients at 18-months, *n* = 92^a^.

Griffiths scales/ sleep spindle features	Subscale A DQ, Foundations of Learning				Subscale B DQ, Language and Communication				Subscale C DQ, Eye and Hand Coordination				Subscale D DQ, Personal-Social-Emotional				Subscale E DQ, Gross Motor				GD DQ, General development			
	Spearman’s Rank Correlation		Partial Spearman’s Rank Correlation^b^		Spearman’s Rank Correlation		Partial Spearman’s Rank Correlation^b^		Spearman’s Rank Correlation		Partial Spearman’s Rank Correlation^b^		Spearman’s Rank Correlation		Partial Spearman’s Rank Correlation^b^		Spearman’s Rank Correlation		Partial Spearman’s Rank Correlation^b^		Spearman’s Rank Correlation		Partial Spearman’s Rank Correlation^b^	
	r	p	r	p	r	p	r	p	r	p	r	p	r	p	r	p	r	p	r	p	r	p	r	p
Synchrony (%)	‒0.13	0.21	‒0.15	0.16	‒0.14	0.19	‒0.16	0.15	‒0.23*	0.03	‒0.25*	0.02	‒0.21*	0.05	‒0.24*	0.03	‒0.30*	0.004	‒0.33*	0.001	‒0.24*	0.02	‒0.28*	0.01
Spectral power (µV^2^)	‒0.07	0.52	‒0.10	0.37	0.13	0.23	0.09	0.42	‒0.11	0.31	‒0.17	0.12	‒0.04	0.73	‒0.10	0.37	‒0.13	0.22	‒0.13	0.25	‒0.03	0.76	‒0.09	0.38
Brain Symmetry Index	0.01	0.89	0.04	0.71	0.03	0.78	0.06	0.59	0.02	0.85	0.05	0.62	0.09	0.40	0.14	0.19	0.18	0.08	0.20	0.06	0.05	0.65	0.10	0.36
Duration (s)	‒0.16	0.13	‒0.15	0.17	0.02	0.86	0.03	0.76	‒0.17	0.11	‒0.16	0.14	‒0.23*	0.03	‒0.22*	0.04	‒0.29*	0.01	‒0.32*	0.003	‒0.17	0.11	‒0.15	0.18
Frequency (Hz)	0.03	0.79	0.01	0.92	‒0.02	0.83	‒0.06	0.61	0.09	0.38	0.06	0.55	‒0.02	0.83	‒0.06	0.61	0.09	0.38	0.09	0.40	0.09	0.38	0.06	0.56
Number^c^	‒0.07	0.54	‒0.03	0.78	‒0.15	0.21	‒0.10	0.41	‒0.05	0.69	0.01	0.97	0.04	0.72	0.11	0.36	‒0.08	0.48	‒0.04	0.72	‒0.13	0.25	‒0.07	0.55
Density^c^ (spindles.min^-1^)	‒0.07	0.54	‒0.07	0.57	‒0.04	0.76	0.01	0.91	‒0.11	0.35	‒0.07	0.57	0.002	0.98	0.06	0.60	‒0.11	0.34	‒0.10	0.42	‒0.15	0.19	‒0.12	0.31

^*^Coefficients whose *p-*value < 0.05.

^a^Unless otherwise stated.

^b^Partial Spearman’s Rank Correlations are adjusted for postnatal age at both EEG and Griffiths assessments, gestational age, and sex.

^c^n = 78, infants who fell asleep during EEG recording.

### qEEG: coherence and spectral power

**Fig. 4:**
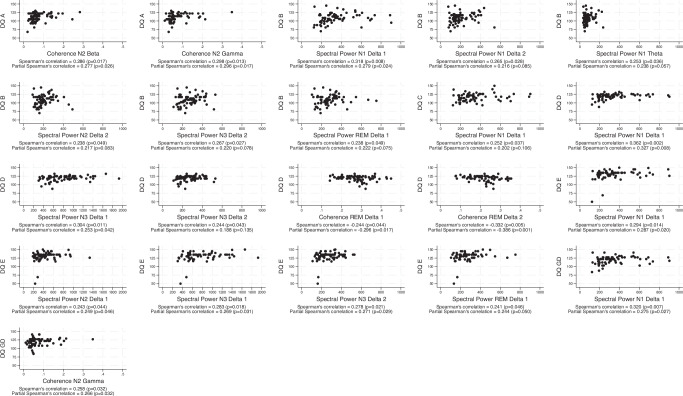
Associations between qEEG features and Griffiths developmental quotients. Scatterplots of the statistically significant Spearman’s correlations. Partial Spearman’s correlations adjusted for gestational age, ages at EEG and Griffiths III assessments, and sex.

Associations between Griffiths developmental quotients at 18-months and, coherence and spectral power at 4-months across the sleep stages are detailed in Supplementary table 1. Figure 4 presents the scatterplots of the statistically significant correlations between coherence and spectral power across the sleep stages, frequency bands and Griffiths developmental quotients (*n* = 69). The majority (15/21) of statistically significant correlations observed were for spectral power in delta frequencies and indicated a small to medium positive association: N1 delta 1 and subscales B (*r* = 0.318, *p* = 0.008), C (*r* = 0.252, *p* = 0.037), D (*r* = 0.362, *p* = 0.002) and E (*r* = 0.294, *p* = 0.014) and general development (*r* = 0.320, *p* = 0.007); N1 delta 2 and subscale B (*r* = 0.265, *p* = 0.028); N2 delta 1 and E (*r* = 0.243, *p* = 0.044); N2 delta 2 and B (*r* = 0.238, *p* = 0.049); N3 delta 1 and D (*r* = 0.304, *p* = 0.011) and E (*r* = 0.283, *p* = 0.018); N3 delta 2 and B (*r* = 0.267, *p* = 0.027), D (*r* = 0.244, *p* = 0.043) and E (*r* = 0.278, *p* = 0.021); REM delta 1 B (*r* = 0.238, *p* = 0.049) and E (*r* = 0.241, *p* = 0.046). During N2, significant small positive correlations were seen in the coherence of higher frequencies and subscale A (beta and gamma) and general development quotients (beta and gamma). Negative small to medium associations were observed for the coherence of low frequencies during REM and subscale D. After adjustment, correlations remained statistically significant for coherence, except for N2 beta and general development; spectral power was no longer statistically significant on B subscale (except on N1 delta 1), nor on the C or D subscales in association with N3 delta 2. Adjusted correlations were small to medium for both coherence and spectral power (Supplementary Table 1).

### Analyses of potential socioeconomic confounders

All participants provided information on maternal level of education and 65/92 participants provided information on household income. No associations were observed between Griffiths and maternal level of education and hence maternal level of education was not a confounder in the relationships between sleep features and Griffiths subscale quotients. Griffiths was associated with household income on subscales B, C, D, and General Development. No sleep spindles or macrostructure features were associated with household income. Associations between income and quantitative EEG (qEEG) were only observed on N3 theta coherence. Partial Spearman’s Rank Correlations between N3 theta coherence and the Griffiths subscales that were associated with household income were calculated (Supplementary table 2); and the conclusions did not differ from the original adjusted analysis (Supplementary Table 1) where no statistically significant relationships were found.

## Discussion

We previously described normative values for sleep macrostructure and sleep spindles in term-born infants at 4 months.^57^ In this study, we investigated associations between sleep EEG features at 4 months and Griffiths’ quotients at 18 months in the same cohort of healthy term-born infants. We analyzed EEG sleep macrostructure features, sleep spindles, and qEEG whole-brain measures: spectral power and interhemispheric coherence. Sleep EEG analysis, particularly sleep spindle synchrony and duration, and delta power, showed correlations with Griffiths III. Intrinsic difficulties in designing and assessing psychometric tests for early infancy and the improvement with age have been discussed and described in the literature,^64–67^ and sleep EEG markers may be a good candidate to help track the trajectory of development in early infancy.

### Sleep macrostructure

A meta-analysis in older children (≥5-year-old) found sleep macrostructure presented very small but statistically significant correlations to cognitive and behavioral aspects. Namely, sleep duration had a positive correlation to cognitive performances and school grades and, a negative correlation to behavioral problems.^15^ A systematic review exploring the association between diurnal napping in younger children (≤5 years-old), in parameters like presence and duration of the nap and difficulty in settling for sleep, and the cognitive performance and behavior found conflicting results and warrants further research.^68^ Still, there is an indication that most studies on naps in children point out their protective role in new learning and on the generalization of word meanings.^69^ Accumulation of learning experiences and consolidation of their abstract representations would, in turn, contribute to establishing and improving developmental skills. Active sleep and REM are associated with synaptic formation, protection and selective pruning in maturing animal models^70,71^ and, activity during NREM may be correlated to synaptic renormalization^24^ and also systematic neuroplastic processes that lead to learning and memory.^43,44^ REM and NREM sleep also show a complementary effect on brain maturation; both appear to play a part in sensorimotor maturation^25,72,73^ and visual learning.^74^ In our study and after adjustment for potential confounders, we found only two statistically significant correlations between the macrostructure properties of the first sleep cycle and future development. Those were positive associations between latency to sleep and the Foundations of Learning subscale and, between the duration of stage N3 and Personal-Social-Emotional subscale. Longer sleep latency may be a limited proxy of resistance to homeostatic pressure and to consequent increased availability in terms of time and predisposition to explore and learn; while deficits in N3 duration may mediate decreased positive mood, as suggested in non-depressed adults,^75^ and by consequence affect emotional regulation and receptivity for social interactions.

### Sleep spindles

Sleep spindle properties change in parallel with brain development.^76^ Like slow wave activity, spindles are associated with learning and memory^44^ and cognitive abilities,^45^ with in vitro studies showing that spindle-like patterns can cause synaptic potentiation or depression.^77^ Sleep spindles reflect both brain anatomical properties^78,79^ and expression of risk-genes associated with some neurodevelopmental disorders,^80^ such as CACNA1I which encodes a subunit of CAV3.3, a T-type calcium channel highly expressed on the thalamic reticular nucleus (TRN) from which sleep spindle expression depends and is simultaneously a risk gene for schizophrenia^81^; consequently, individuals with schizophrenia and their first-degree relatives show sleep spindle alterations.^82,83^

#### Spindle synchrony

Negative correlations were observed between spindle synchrony and General Development and subscales of Eye and Hand Coordination, Personal-Social-Emotional and Gross Motor skills (subscales C, D and E). Spindles are predominantly local phenomena.^21,84,85^ In adults, scalp EEGs have shown that hemisphere-specific spindle activity increases are related to previous procedural learning^35^ and are correlated with overnight verbal memory encoding.^39^ Also, abstract reasoning abilities appear to be associated with side predominance of spindle activity.^17^ As described by Louis et al. there is a trend of decreasing synchrony from 3 to 6 months (calculation of asymmetry in Louis et al. study was based on the interval between the middle of two contralateral spindles being more than 2 seconds).^47^ As such, the observations on spindle synchrony may also reflect its maturational aspect.

#### Spindle duration

In infants after 3 months and toddlers, there is a downward trend in spindle duration linked to age,^27,47,86,87^ which may indicate that the shortening of sleep spindles is associated with the normal course of brain maturation at this age. A multiplicity of factors induces spindle termination and thus determines spindle duration. Several of these factors may work together,^88^ some related to connectivity and synchronization between cortical and thalamic structures. Corticothalamic inputs support thalamic propagation and influence the rhythmicity of thalamic spindle oscillations.^89^ In this manner, desynchronization between thalamic and cortical networks during the waning phase of spindles progressively depolarizes thalamic neurons, hindering the generation of a new spindle oscillation cycle.^90^ In addition, both the strength and spread of the thalamocortical and corticothalamic networks are correlated with spindle duration.^85^ The thalamocortical functional connectivity to the sensorimotor area decreases during infancy^91^ possibly associated with increased functional network specialization, potentially affecting spindle duration. In this study, we found a negative correlation between spindle duration and gross motor skills development, supporting the notion that decreases in sensorimotor neural connectivity in infancy may be associated with the process of progressive specialization.^91–93^

#### Sleep spindle associations with motor and personal-social-emotional skills

Altered sleep spindles have been reported in cerebral palsy, a condition characterized by motor deficits^94^; also, interhemispherical spindle power asymmetry in perinatal unilateral brain injury in infancy has a high positive and negative predictive power for later development of unilateral cerebral palsy.^95^ Furthermore, on an early night study of 6-month-old infants Jaramillo et al. found a positive association between spindle density above 13.5 Hz and mean spindle frequency, and gross motor skills reported by parents using Ages and Stages Questionnaire at 24 months after adjusting for age and sex.^96^ Different age groups and methodologies may justify the different observations between the latter and the present studies. However, our study builds on evidence highlighting the role of sleep spindles in infancy and the maturation of motor skills.^25^

In 5-year-old children, nighttime number and frequency of spindles are positively correlated with stress-coping strategies, personal and social behavior,^97^ number of spindles is also predictive of emotional and behavioral competencies one year later.^98^ In contrast, studies on spindles in younger children during naps or partial night sleep recordings found non-significant results on the association between the spindle features analyzed and scales evaluating behavioral and social skills. Page et al. reported no association between spindle frequency, amplitude, duration and density in children from 12 to 30 months and their current scores using Vineland Adaptive Behavior Scales–Second Edition.^27^ Jaramillo et al., found no association between spindles at 6 months, namely density above 13.5 Hz and mean spindle frequency, during early NREM night sleep and parents reported personal-social skills at the time of recording and at 12 and 24 months.^96^ In the present analysis, we found that spindle duration and synchrony at 4 months were predictive of the Personal-Social-Emotional quotient at 18 months. Infancy and toddlerhood are marked by successive milestones in self-awareness, social and emotional development domains.^99,100^ The default mode network has been correlated with self-referential processes^101,102^ and social competencies.^103,104^ The infancy period coincides with the emergence of functional connectivity within default mode networks^105^ and between the default mode networks and the thalamus^91^ which may explain the associations of sleep spindles with later observed behavior.

### qEEG

#### Delta spectral power

Spectral power was the qEEG measure that showed the highest number of significant correlations to Griffiths developmental quotients at 18 months. When evaluating magnitude across the different EEG frequency bands through spectral power analysis, quotients of General Development and subscales B (Language and Communication), D (Personal-Social-Emotional) and E (Gross Motor) were positively associated with delta frequencies after adjustment. In a study by Kim et al. when 0.1–4 Hz microelectrode-detected waves were divided into two classes based on the amplitudes of the up- and preceding down-states, they were found to have distinct and opposing roles,^106^ compatible with progressive down-selection of synapses,^13,106^ or with synaptic strengthening.^106,107^ According to the sleep homeostatic hypothesis, synaptic strength increases with wakefulness duration and learning activities. It has, however, an associated energy cost that leads to a need for renormalization through net synaptic down-selection, ideally during brain “offline” periods.^13^ In an incompletely myelinated brain, with a high level of redundant synapses such as in the maturing brain, net synaptic down-selection may promote brain energy efficiency.^14^ Additionally, slow oscillations are also believed to facilitate temporal coupling with other oscillations such as sleep spindles and sharp wave ripples,^108,109^ contributing to the transfer of particular memory traces from the hippocampus to the neocortex^44^ (see Mason and Spencer^110^ for discussion on the active system consolidation in infancy and childhood). In the pediatric population, slow wave activity is affected by the prior duration of wakefulness, previous awakening stimuli exposure, and brain maturation^111^ including myelination.^28,33^ The daily slow wave activity gains, characteristic of childhood, are thought to assist the consolidation of abstract representations of memory traces and to allow generalizable consolidation of new learning in early development.^111^ The continued influence of incremental slow wave activity may facilitate a multitude of complex skill learning.^26^ Two subscales were not associated with delta power, those were Foundations of Learning and Eye and Hand Coordination (subscales A and C). In line with our findings on subscale A, another developmental study found no association with the current cognitive skills of infants involved.^112^ In light of the described function of slow waves, it may seem counterintuitive that Foundations of Learning is not associated with delta power. However, subscale A does not necessarily inform on long-term memory abilities, focusing instead on abilities to explore and receive new information such as creative play and executive function. Subscale A maturation may be better reflected in parameters like coherence levels^113^ as also demonstrated in this study.

In studies assessing 8-month-olds or young children, delta amplitude over frontal and occipital areas was positively associated with current fine motor abilities.^27,112^ Despite these observations, our study showed no association between delta power at 4 months and later Eye and Hand Coordination levels, possibly indicating that the previous findings of associations between fine motor skills and delta activity are specific to frontal and occipital regions, not extending to whole-brain analysis or to future development. We performed many correlation analyses in this study which increases the probability of false positives. We note however, all correlations were concentrated towards delta power, making the observations consistent and these associations are expected to be stronger amongst defined neurodiverse cohorts.

#### Interhemispheric coherence

Functional connectivity as measured by EEG during resting-state,^114^ including sleep,^51,52^ evolves across development. This evolution in early life is concurrent with structural and sleep pattern development, although, the nature of these interactions is still poorly understood.^31^ Neonatal EEG connectivity changes across sleep states are associated with visual performance at 2 years of age, suggesting that sleep functional connectivity may predict later development. Associations between sleep EEG coherence and cognitive abilities were also found in older pediatric populations, where maturational changes in NREM sigma intrahemispheric coherence were associated with cognitive aspects such as attention.^115^ Additionally, cohorts of neurodiverse children and adolescents may present sleep coherence alterations relative to neurotypical peers.^116^ While genetics may play an important role in determining sleep EEG connectivity, it can be partially modulated through family factors and interventions through co-regulatory maternal emotional conditioning and physical proximity such as calm maternal interactions and co-sleeping arrangements^117–119^ which have the potential to improve aspects such as social-relatedness, attention and neurodevelopment.^120^

After controlling for gestational and postnatal age at the time of the appointments and sex, we noted two main trends amongst the significant results on interhemispheric coherence at 4 months. One was the interhemispheric coherence of fast rhythms which were positively correlated with subscale A at 18 months during N2. Subscale A assesses features related to learning aspects such as skills for learning, ways of thinking, working memory, and play.^5^ Tasks requiring executive function^113^ and associative learning^121^ may produce functional changes in connectivity measured by EEG coherence. Neuronal coherence is hypothesized to reflect neuronal communication^122^ with the potential for changes in synaptic plasticity.^123^ Adding to these observations, our study shows that ‘offline’ aspects of global interhemispheric connectivity may partially indicate skills for learning at 18 months.

The second trend we observed was increased delta frequency interhemispheric disconnection during REM for infants who performed better on subscale E in early childhood. This subscale focuses on personal-social-emotional aspects. Infants who score higher on this scale demonstrate higher independence, higher levels of empathy and tempered humor and seek more social interactions.^5^ Sleep has been associated with mood and emotional processing; its deprivation is related to decreased social and intrapersonal functioning.^124,125^ Evidence suggests REM plays a critical role in sleep emotional processing, although there are discrepancies between studies regarding the direction of this modulation.^126^

Despite the associations observed between coherence and future development, it is important to note that, due to the large number of correlations calculated, statistically significant results may be false positives (type I error). Thus, the global interhemispheric coherence measured in this study should be regarded with caution.

### Socioeconomic factors

Household income and highest level of maternal education were investigated as potential confounders in the relationships between the sleep features and Griffiths III quotients. Maternal education was not a confounder as it was not associated with Griffiths III quotients and hence we did not adjust for it in any analysis. We noted that although household income was associated with Griffiths III quotients at 18 months, the association between sleep features and Griffiths III remained unaltered when adjusted for household income (Supplementary table 2). This analysis had less than three-quarters of the participants from the original qEEG analysis, as only 50/69 participants provided income information which may have limited results. However, it should be noted that parental socioeconomics and alterations to this status influence childhood cognitive scores^127^. Low childhood cognitive scores, in its turn, impact the risk for adversities later in life such as low education, income and higher risk of depression.^127^ Early identification of risk for lower cognitive outcomes may allow psychosocial intervention with the potential of  improving cognitive outcomes up to adulthood,^128,129^ better psychosocial outcomes,^129^ education achievement^129^ and job situation.^130^

### Summary

In summary, sleep spindle synchrony and duration, as well as, delta power present consistent small to medium associations with development at 18 months. Together, sleep spindles and delta power were associated with 4/5 Griffiths’ subscales (the exception was Foundations of Learning) and general development quotients at 18 months. Macrostructure features and interhemispheric coherence, as analyzed here, should be interpreted with caution due to the possibility of type I error.

### Strengths/limitations

This paper analyses a diverse range of sleep EEG biomarkers in term-born infants and their association with future development. This is one of the largest cohorts relating future cognitive ability to EEG sleep in healthy infants.

We recorded sleep in the sleep lab, which allowed us to control for environmental stimuli that could interfere with sleep and thus provided standardized conditions to the participants. However, due to practical logistic considerations, we did not perform an adaptation nap. The first sleep in a lab correlates with lower sleep efficiency and higher proportions of light sleep in children compared to subsequent sleeps.^131^ In adolescents at least, sleep spectral power^132,133^ and sleep spindles parameters^134,135^ show good individual stability over consecutive nights, except for duration and density of slow spindles.^136^ Slow spindles are a type of spindle that emerges in older populations than our participants when their EEGs were recorded,^137^ and for this reason, this factor will not affect our observations. As noted by Beaugrand et al.^34^ a desirable EEG marker of development should allow a feasible application to clinical settings. Therefore, studying sleep markers from the first sleep recording presents an advantage for adapting protocols to clinical settings.

Our objective was to examine a diverse range of EEG biomarkers and assess which features better predict development. However, multiple tests do increase the likelihood of type 1 error. Despite that, we observed that statistically significant features gravitate towards sleep spindle synchrony and duration and delta spectral power, making these promising biomarkers for predicting development. These biomarkers may facilitate early identification of risk for adverse outcome in infants, which would enable early and targeted intervention.

There is a high prevalence of well-educated mothers and high-income families among those who consented to participate in this study. Additionally, the median (IQR) values for maternal EPDS and maternal-infant attachment indicate a low likelihood of depression and high attachment in the study group (Table 1), which may also enhance aspects of developmental outcomes in our cohort.^138–140^ A possible limitation to this study is the underrepresentation of less favorable contexts.

We used Spearman’s rank correlations in our analysis because some of the examined correlations did not meet the assumptions of normality and linearity required for Pearson’s correlation. Spearman’s rank correlation assesses monotonic relationships rather than linear ones. We refer the reader to the scatterplots present in Fig. 4 for a visual interpretation. This is an initial study and future studies should include multivariable analysis of the sleep parameters identified in this study as being associated with neurodevelopment at 18 months.

Most of the statistically significant results showed small to medium associations, indicating that the predictive ability of the sleep parameters at 4 months to determine Griffiths III neurodevelopmental outcomes obtained approximately 14 months later is weak. Given that neurodevelopment is influenced by ongoing environmental influences, and that the diagnostic accuracy of psychometric tests improves with age, we plan to continue following this cohort until school-going age (5-year-old) to investigate the strength of the correlations between the sleep patterns at 4 months and later neurodevelopmental outcomes.

Future studies are needed to validate the present findings and to investigate the sleep EEG features identified here on specific developmental conditions at early ages.

## Conclusions

Our study has shown that sleep, and in particular EEG sleep spindles and delta power, may be a useful biomarker of development in early childhood. Future studies aimed at identifying potential atypical development to facilitate early intervention, should consider the sleep biomarkers analyzed here.

## Supplementary information

Supplementary information - CONSORT flow diagram

Supplementary table 1 - Association between qEEG parameters and Griffiths quotients at 18 months, n=69.

Supplementary table 2 - Association between Griffiths III quotients and sleep features that are co-associated with household income, n=50.

## Data Availability

It is not possible to share the datasets for the current study. The clinical data is collected under a written proxy consent from the participants’ guardians/parents, which did not include permission for sharing or open data. To be allowed to share this data under Irish Health Research Regulations we are required to re-consent families or to obtain approval by the Health Regulation Consent Declaration Committee.
