# Supplementary material for: Infant sleep EEG features at 4 months as biomarkers of neurodevelopment at 18 months
Source: Pediatr Res. 2025 Feb 20;98(4):1474–85. doi: 10.1038/s41390-025-03893-6 (PMC12549339; doi:10.1038/s41390-025-03893-6)
Supplement: Supplementary file 1 — Supplementary information - CONSORT flow diagram [file 41390_2025_3893_MOESM1_ESM.docx]

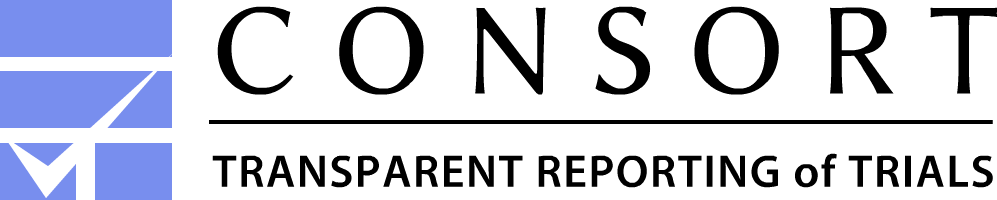


**CONSORT 2010 Flow Diagram**

## Follow-Up

Allocated to control group (care as usual) (n=204)

## Allocation

Randomized (n=408)

Excluded (n=1,892)

♦  Not meeting inclusion criteria or declined to participate

Assessed for eligibility (n=2,300)

## Enrollment

Lost to follow-up at 4 months sleep EEG assessment (n=106)

Reasons:

- Parents withdrawal consent. Motives pointed:
  - Unhappy with group allocation;
  - Found filling out sleep diary/app time consuming;
  - Illness in family;
  - Unable to attend the 4-month appointment at the requested age;
- Parents unreachable through calls or texts.

Discontinued participation after EEG assessment (n=2)

Reasons:

- Grossly abnormal EEG (periventricular leukomalacia) (n=1);
- Failed to fall asleep (n=1).

Lost to follow-up at 18 months Griffiths assessment (n=4)

Reason: Unable to attend the 18-month appointment at the requested age.

Allocated to intervention (n=204)

[Intervention group not included in the present analysis)

*Continues on the next page*

## Analysis

Sleep spindles analysis:

- Analysed for spindle number and density (n=78/92)
  Excluded from analysis (n=14)
  Reason: Infants fell asleep before onset of recording
- Analysed for all other sleep spindle parameters: (n=92/92)

Excluded from analysis (n=0)

Sleep macrostructure and qEEG analysis:

- Analysed for general parameters (n=69/92)
  Excluded from analysis (n=23)
  Reason: Infants who did not have a complete first sleep cycle recorded

Exceptions:

- Analysed for total nap duration (n=78/92)
  Excluded from analysis (n=14)
  Reason: Infants fell asleep before onset of recording
- Analysed for latency to REM (n=67/92)
  Excluded from analysis
  Reason:
   -Infants who did not have a complete first sleep cycle recorded (n=23).
   -Infants who met criteria for staging REM before N2 (n=2)
