# Supplementary material for: Infant sleep EEG features at 4 months as biomarkers of neurodevelopment at 18 months
Source: Pediatr Res. 2025 Feb 20;98(4):1474–85. doi: 10.1038/s41390-025-03893-6 (PMC12549339; doi:10.1038/s41390-025-03893-6)
Supplement: Supplementary file 3 — Supplementary table 2 - Association between Griffiths III quotients and sleep features that are co-associated with household income, n=50. [file 41390_2025_3893_MOESM3_ESM.pdf]

Supplementary table 2 - Association between Griffiths III quotients and sleep features that are co-associated with household income, n=50.

Griffiths III Measures	Sleep stage	qEEG feature	Frequency band	Partial Spearman's Rank Correlation[1]	
				r	p-value
Subscale B DQ, Language and Communication	N3	Coherence	Theta	0.003	0.985
Subscale C DQ, Eye and Hand Coordination	N3	Coherence	Theta	0.089	0.560
Subscale D DQ, Personal-Social-Emotional	N3	Coherence	Theta	0.208	0.171
GD DQ, General Development	N3	Coherence	Theta	0.076	0.622

[1]Partial Spearman's Rank Correlations are corrected for postnatal age of both EEG and Griffiths at 18-months assessments, gestational age, sex and household income.
